# Supplementary material for: Embodied metaphor in communication about lived experiences of the COVID-19 pandemic in Wuhan, China
Source: PLoS One. 2021 Dec 30;16(12):e0261968. doi: 10.1371/journal.pone.0261968 (PMC8718003; doi:10.1371/journal.pone.0261968)
Supplement: S1 Appendix — (DOCX) [file pone.0261968.s001.docx]

**Appendix A. Pandemic metaphor category**

| **Metaphor category** | **Frequency** | **Ratio** | **Examples** |
| --- | --- | --- | --- |
| image metaphor | 95 | 18.4% | During the pandemic, if we are little stars, our country is the sun. It is a cluster of flames and we are the sparks. |
| motion | 33 | 6.4% | My heart was going to jump out of my body. |
| war | 30 | 5.8% | When we fought on the frontline…. We were those who fought a battle while they were the one who backed up us. They provided us with support to make sure we encounter no more troubles. |
| sense | 24 | 4.7% | I used to think that the relationship between people in this society might be very cold, but now I believe that warmth exists in this world and in this society because of them. |
| symbolic metaphorical enactment | 21 | 4.1% | I’m petrified of death. So before I entered the COVID-19 ward, I changed the profile photo of WeChat to the photo of Dr. Zhong Nanshan, hoping for his blessing. |
| darkness and light | 20 | 3.9% | Initially, Wuhan was slowly invaded by darkness. But at the same time, a faint light began to shine every corner of the city and the darkness was dispersed by the gathering of the light. |
| spatialization | 19 | 3.7% | Death means that the soul rises to heaven. |
| life and death | 18 | 3.5% | Being discharged from the hospital, I felt like I was being reborn. |
| integrative behavior | 17 | 3.3% | Most COVID-19 patients were extraordinarily anxious. When they were admitted to the hospital, they constantly looked for doctors and nurses and kept talking to us about the illness. Why? That’s because they might feel safe with us around. |
| container | 17 | 3.3% | You fall into a completely panic because you never know why this is happening to you. |
| animal | 14 | 2.7% | We were the laboratory mice in all of the treatments the doctors offered to us. It felt like being caged and you are a laboratory mouse. |
| games, chance, sport | 14 | 2.7% | It is cruel…it’s a game of Russian roulette. I mean you don't know if you're the next one to be chosen. It is the case for those who have been infected with COVID-19 and died. |
| violence and impact | 11 | 2.1% | Well, maybe the death of the old man stroke me a bit. |
| time | 11 | 2.1% | The citizens of Wuhan rushed to purchase the necessities. It seemed as if we were living in a time of famine. Things looked desolate. |
| temperature | 10 | 1.9% | When sad news surged into you, you may feel so cold and tremble with fear as if the end of the world was coming. |
| animacy | 10 | 1.9% | We cannot relax because we have no idea when COVID-19 will attack Wuhan again in retaliation. |
| physical injury | 9 | 1.7% | The traffic was paralyzed. |
| color | 9 | 1.7% | When I was quarantined at home, I felt that the sky and the world outside were grey and dark. |
| closeness and distance | 9 | 1.7% | It is indelibly imprinted in my brain because I have never before imagined the feeling of staying so close to death. |
| weight | 8 | 1.6% | Everyone felt heavily-laden at the outbreak of COVID-19. |
| family | 8 | 1.6% | As for the image of the country, he is an excellent parent but not perfect. |
| machine | 8 | 1.6% | It seemed that the whole city had stopped. It was like a giant gear that had stopped working. It was broken down and tons of people rushed to repair it. We hoped that it could function well again. |
| not feeling part of the world | 8 | 1.6% | I hoped that I was just in a dream and I would wake up soon. It seemed like a nightmare. I really hoped that I could wake up to end the nightmare soon. |
| presence, accompany and absence | 8 | 1.6% | I was petrified, at the beginning, of treating COVID-19 patients, feeling that I was surrounded by viruses. |
| plant | 7 | 1.4% | Sometimes our life is like a tree, a sturdy tree that cannot be vibrated. It might be shaken slightly from trauma, because the life, for some people, is tenacious. |
| up and down | 6 | 1.2% | My mood fluctuated in the face of death. But gradually, I packed and arranged the corpses skillfully, handed them to the undertakers, and resumed my work. |
| explosion | 6 | 1.2% | It (reading the news about the pandemic) seems like watching a volcano that keeps erupting. Sometimes it is grievous or sometimes venial. |
| pressure | 5 | 1.0% | The pressure was an inflated balloon that might explode sooner or later. |
| trials, law | 5 | 1.0% | I think that in terms of probability, I am one of those who are sentenced to death. |
| liquid-based metaphor | 4 | 0.8% | Everyone sticks together like a river. Well, it’s like a…I mean we gather together and the river surges forward filled with vitality. |
| inside and outside | 4 | 0.8% | The city went into lockdown quickly, preventing the virus from getting out. |
| physical sensation and pain | 4 | 0.8% | I felt that my body had been hollowed out when I left the COVID-19 ward. |
| building | 4 | 0.8% | The rehabilitation center is a protection station. |
| fairness, justice, chance | 4 | 0.8% | I felt helpless. Why should I be the one that was infected? I only went to Hankou on that day and why should I be the one that was infected? |
| journey | 4 | 0.8% | The journey of our medical treatment was quite tortuous. |
| finding and losing | 3 | 0.6% | Death means that you might unconsciously lose everything. |
| pushing and pulling | 3 | 0.6% | Then they pulled us up or brought us hope. |
| link, bound, and connection | 4 | 0.8% | I consider all the citizens in Wuhan as a rope. We are united. |
| crumbling, breaking,  falling apart | 3 | 0.6% | Just like being in the flood season right now, death, for some families, could be a flash flood, mudslide and landslide because your death will bring major changes to your family. |
| carry | 3 | 0.6% | I admire the volunteers very much because they carry the danger. They carry the danger for us. |
| going back and forth | 2 | 0.4% | The lockdown of the city made me feel like going back to the year of SARS. The teachers gave each of us a bucket and we wiped the tables every day. |
| depth | 2 | 0.4% | An uncomfortable feeling existed in the depth of my heart when I heard that a lot of people had paid or or been sacrificed for this. |
| nature | 2 | 0.4% | The doctors and the volunteers are timely rain, I think so. |
| mixed metaphor | 2 | 0.4% | Death is a returning. It is like a deep sleep. It is like dust fading away in the air. It is like a drop of water flowing into the sea, disappearing forever. |
| play | 2 | 0.4% | During the outbreak of COVID-19 in China, we were all actors involved in the pandemic. But when we watched the news about the outbreak in other countries, we were the audience. |
| conduit metaphor | 2 | 0.4% | I was very sad and felt that my heart was plugged up at the death of the patients. |
| agency | 2 | 0.4% | The Gang is like a top that will move only when you twitch it. |
| guardian | 1 | 0.2% | In my mind, the image of the country is like an angel with wings. The wings holding us are quite warm, but their backs drip with blood. |
| struggling | 1 | 0.2% | It’s like the game called *Chi Ji*. Everybody struggles inside the circle of viruses and you don't know when you’re going to fall apart. |
| 49 types | 516 | 100% |  |

Note: As one metaphorical utterance may encompass multiple metaphor categories, the total frequency of metaphorical instances was 516, rather than 370.
