# Supplementary material for: Embodied metaphor in communication about lived experiences of the COVID-19 pandemic in Wuhan, China
Source: PLoS One. 2021 Dec 30;16(12):e0261968. doi: 10.1371/journal.pone.0261968 (PMC8718003; doi:10.1371/journal.pone.0261968)
Supplement: S2 Appendix — (DOCX) [file pone.0261968.s002.docx]

**Appendix B.** **Topics of the pandemic metaphors**

| **Topic** | **Frequency** | **Ratio** |
| --- | --- | --- |
| life and death | 37 | 10.0% |
| combat COVID-19 | 30 | 8.1% |
| emotional state at the outbreak of COVID-19 | 27 | 7.3% |
| image of country's role | 24 | 6.5% |
| emotional state at lockdown of Wuhan city | 21 | 5.7% |
| image of lockdown of the city | 19 | 5.1% |
| image of COVID-19 | 18 | 4.9% |
| unselfish service, bravery | 16 | 4.3% |
| virus | 15 | 4.1% |
| emotional state at being infected by COVID-19 | 9 | 2.4% |
| emotional state of being isolated | 9 | 2.4% |
| image of doctors and social workers | 9 | 2.4% |
| the social relationship between people | 8 | 2.2% |
| patient's mental state (in hospital/at home) | 7 | 1.9% |
| behavior-related mental anxiety | 6 | 1.6% |
| COVID-19 patient leaves his/her child | 6 | 1.6% |
| dream, anxiety and fear during COVID-19 | 6 | 1.6% |
| witnessing the death of patients | 5 | 1.4% |
| extreme anxiety during COVID-19 | 5 | 1.4% |
| emotional state at combatting COVID-19 | 4 | 1.1% |
| emotion state of death during COVID-19 | 4 | 1.1% |
| emotional state at leaving hospital | 4 | 1.1% |
| emotional state of being in the hospital | 4 | 1.1% |
| image of medical staff | 4 | 1.1% |
| outbreak of COVID-19 | 4 | 1.1% |
| worry, fear | 4 | 1.1% |
| increasing number of COVID-19 patients | 3 | 0.8% |
| being infected by COVID-19 | 3 | 0.8% |
| social work | 3 | 0.8% |
| bad experience of city during COVID-19 | 2 | 0.5% |
| comments on officials | 2 | 0.5% |
| situation in the community | 2 | 0.5% |
| COVID-19 and the nation | 2 | 0.5% |
| emotional state in doing social work | 2 | 0.5% |
| end of quarantine and leaving the hotel | 2 | 0.5% |
| image of hospital | 2 | 0.5% |
| panic at home | 2 | 0.5% |
| patients' despair | 2 | 0.5% |
| reaction to COVID-19 | 2 | 0.5% |
| rumor of lack of essentials; crazy shopping | 2 | 0.5% |
| transportation | 2 | 0.5% |
| fragileness of life | 2 | 0.5% |
| exhaustion and physical pain | 2 | 0.5% |
| serious situation of the COVID-19 pandemic | 2 | 0.5% |
| anger at the delayed hospitalization | 1 | 0.3% |
| anger with the service | 1 | 0.3% |
| being tired | 1 | 0.3% |
| emotional state at negative news | 1 | 0.3% |
| emotional state at waiting for test result | 1 | 0.3% |
| emotional state of being healthy | 1 | 0.3% |
| emotional state of being safe | 1 | 0.3% |
| emotional state of business as usual | 1 | 0.3% |
| emotional state of going out | 1 | 0.3% |
| emotional state of going to patients' room | 1 | 0.3% |
| emotional state of medical service | 1 | 0.3% |
| end of the COVID-19 pandemic | 1 | 0.3% |
| examining COVID-19 patients | 1 | 0.3% |
| feeling after work | 1 | 0.3% |
| impression of isolation | 1 | 0.3% |
| Industries and economy | 1 | 0.3% |
| In-patient room | 1 | 0.3% |
| medical treatment of COVID-19 patients | 1 | 0.3% |
| feeling moved,being excited | 1 | 0.3% |
| mutual support | 1 | 0.3% |
| feelings of nervousness | 1 | 0.3% |
| pressure during COVID-19 | 1 | 0.3% |
| relationship between doctors and patients | 1 | 0.3% |
| relationship between humans and nature | 1 | 0.3% |
| telling others about the COVID-19 infection | 1 | 0.3% |
| united situation among people | 1 | 0.3% |
| **70 topics** | **370** | **100%** |
